# A Dash of Virtual Milk: Altering Product Color in Virtual Reality Influences Flavor Perception of Cold-Brew Coffee

**DOI:** 10.3389/fpsyg.2020.595788

**Published:** 2020-12-03

**Authors:** Qian Janice Wang, Rachel Meyer, Stuart Waters, David Zendle

**Affiliations:** ^1^Department of Food Science, Aarhus University, Aarhus, Denmark; ^2^Department of Computer Science, University of York, York, United Kingdom

**Keywords:** virtual reality, augmented virtuality, mixed reality, coffee, food color, consumer perception

## Abstract

It is well known that the appearance of food, particularly its color, can influence flavor perception and identification. However, food studies involving the manipulation of product color face inevitable limitations, from extrinsic flavors introduced by food coloring to the cost in development time and resources in order to produce different product variants. One solution lies in modern virtual reality (VR) technology, which has become increasingly accessible, sophisticated, and widespread over the past years. In the present study, we investigated whether making a coffee look milkier in a VR environment can alter its perceived flavor and liking. Thirty-two United Kingdom (UK) consumers were given four samples of black cold brew coffee at 4 and 8% sucrose concentration. They wore VR headsets throughout the study and viewed the same coffee in a virtual setting. The color of the beverage was manipulated in VR, such that participants saw either a dark brown or light brown liquid as they sipped the coffee. A full factorial design was used so that each participant tasted each sweetness x color combination, Participants reported sweetness, creaminess, and liking for each sample. Results revealed that beverage color as viewed in VR significantly influenced perceived creaminess, with the light brown coffee rated to be creamier than dark brown coffee. However, beverage color did not influence perceived sweetness or liking. The present study supports the role of VR as a means of conducting food perception studies, either to gain a better understanding of multisensory integration, or, from an industry perspective, to enable rapid product testing when it may be time-intensive or costly to produce the same range of products in the real-world. Furthermore, it opens potential future opportunities for VR to promote healthy eating behavior by manipulating the visual appearance of foods.

## Introduction

We eat with our eyes (Delwiche, 2012). Vision plays a fundamental role in the eating experience, as it evolved to facilitate foraging and feeding (Gehring, 2014; Spence et al., 2016). The visual appearance of food not only stimulates our appetite (Wang et al., 2004), but also influences our anticipated and actual eating pleasure (Hurling and Shepherd, 2003). Of all the visual aspects, the color of the food we eat has been especially studied in relation to flavor perception.

### Color-Flavor Interaction

Crossmodal correspondences between color and flavor identification/perception have been well-documented in research (see Spence et al., 2010, and Spence, 2015 for reviews). Over the past 50 years, studies have shown that people consistently associate each of the basic tastes with specific colors; for instance, sweetness with red/pink, sourness with green/yellow, saltiness with blue/white, and bitterness with dark colors like black and deep blue (e.g., Favre and November, 1979; Spence, 2015; Woods and Spence, 2016). Such color–taste/flavor pairs are arguably the result of associative learning, when one learns to associate a specific color with a specific gustatory cue through repeated exposure. This would explain cross-cultural differences in color-taste/flavor pairings, for instance, light blue being associated with mint flavor for Taiwanese participants but raspberry for United States (US) participants (Shankar et al., 2010). Interestingly, new color-taste associations can be learned even with relatively brief exposure periods (e.g., four training sessions, Higgins and Hayes, 2019).

An extensive body of research has demonstrated how food color can drive perceptual changes in the tasting experience. On a basic level, increasing the color intensity of foods and drinks has led to higher taste/flavor intensity ratings (e.g., Johnson and Clydesdale, 1982; Norton and Johnson, 1987; Calvo et al., 2001). When it comes to specific tastes, red-colored drinks have been found to enhance sweetness detection (Johnson and Clydesdale, 1982), expectations (Wei et al., 2012), and perceived intensity (Pangborn, 1960; Clydesdale et al., 1992; Lavin and Lawless, 1998; Hidaka and Shimoda, 2014). Food color can also interfere with flavor identification, such as shown by Zampini and colleagues with lime‐ and orange-flavored beverages colored green or orange (Zampini et al., 2007). With a more complex beverage, Wang and colleagues have demonstrated recently that participants used rosé wine aroma and flavor terms to describe a white wine that had been dyed pink to match the color of an actual rosé wine (Wang and Spence, 2019).

More recently, human-computer interaction research has incorporated technology to alter the sensory input of given foods. For instance, Ranasinghe and colleagues created two such beverage delivery systems, the Virtual Lemonade and the Vocktail. The Virtual Lemonade uses colored LED lights to overlay a color on plain water, combined with electrically stimulated sour taste sensations on the user’s tongue (Ranasinghe et al., 2017a). The Vocktail goes one step further, by combining colored lights and electrically induced tastes with scent delivery, to digitally alter the flavor of a given drink (Ranasinghe et al., 2017b).

### The Use of Virtual Reality in Food-Related Research

Virtual Reality can be defined as a type of human-computer interaction where one interacts with a three-dimensional computer-generated environment presented *via* a stereoscopic head mounted display which tracks the location of the user’s head (Crofton et al., 2019). Given the increasing popularity of VR, several studies have been conducted in the last decade to understand whether VR is suitable for use in food research.

The potential usefulness of this kind of research is supported by emerging evidence that the food-related decisions that people make in VR appear to replicate decisions that they make in the real-world. For example, Ung and colleagues exposed participants to both real-world and VR buffets, in order to investigate whether the nutritional value of foods selected by participants in VR paralleled the value of the foods that they selected in real-world contexts (Ung et al., 2018). There was a strong correlation between the nutritional values of food that participants served themselves in VR and real-world environments (*r* ≥ 0.75). Similarly, participants exposed to both real-world and VR store environments made similar cereal product selection decisions in VR as in the physical environment (Siegrist et al., 2019). In the same vein, researchers placed parents in VR and real-world environments, in order to investigate whether the portions of a pasta-based meal that they chose for their children in VR were similar to the portions that they selected in the real-world (Persky et al., 2018). Results showed that there was a strong relationship between the portions that parents served in VR, and in the real-world. For example, a correlation of *r* = 0.822 was observed between the amount of real apple juice that a parent served and the amount of VR apple juice that they served. In terms of consumer testing, a comparison of beer tasted in a laboratory, in a real pub, or in a variety of immersive environments ranging from projection walls to VR headsets demonstrated that repeatability of hedonic scores for beer was better both in the real pub and in the immersive environments, compared to the central lab testing site (Sinesio et al., 2019). These findings suggest that the use of VR in food research is a useful and meaningful method as participants have been found to treat food the same way in both VR and the real-world. This is especially important given the increasing use of VR in food disorder therapy, either by altering participants’ self-image or by introducing virtual food cues (see Clus et al., 2018, for a review).

Furthermore, a nascent body of literature examines how digitally introduced colors may influence taste perception. For example, Huang and colleagues examined how displaying different colors of tea (red or green) in VR influenced individuals’ subsequent taste ratings of drinks in the real-world (Huang et al., 2019). Results of this study largely did not find any overall effect of color. However, the authors did suggest that there were some color-induced taste rating differences driven by individual color–food associations. More recently, Ammann et al. (2020) assessed whether changing the color of foods (two juices and a piece of cake) shown in VR influenced flavor identification. They demonstrated that seeing modified product colors in VR did in fact negatively impact flavor identification, and that flavor identification performance was not significantly different when participants did the study in VR vs. in real life, using food-coloring-modified products. Finally, even food-extrinsic changes in virtual environment have been shown to influence taste evaluations of different foods, such as a grenadine-based beverage tasting sweeter when consumed in a sweet-congruent environment compared to a bitter-congruent environment (Chen et al., 2020), or when cold brew coffee tastes sweeter when consumed in an environment with pleasant color and music, compared to one with unpleasant color and music (Nivedhan et al., 2020).

Color-induced gustatory effects have been found in studies using augmented reality (AR) technology. In contrast to VR, AR projects digital information (typically visual imagery) onto the physical world (Crofton et al., 2019), therefore plausibly providing a greater degree of realism in the entire eating scenario. For instance, Okajima and colleagues have constructed an AR “food changer” system to identify and modify the appearance of food *via* a sophisticated computer vision algorithm, either using a projector (Nishizawa et al., 2016) or a head-mounted display (Ueda and Okajima, 2019; Ueda et al., 2020). The findings of their experiments, which changed the color, saturation, and visual texture of various foods, found that there was a correlation between the color saturation and the rated sweetness in cake (Nishizawa et al., 2016); the mouthfeel, greasiness, and deliciousness of sashimi can be altered by its visual texture and color (Ueda and Okajima, 2019) and the moistness and deliciousness of sponge cake, as well as the watery taste in ketchup, can be altered by dynamically modifying the luminance distribution of the foods. However, the studies are limited by small sample sizes (four participants in the cake study, 12 participants in the sashimi study, and 13 participants each in the sponge cake and ketchup study).

In a demonstration of altering both color and smell, Narumi and colleagues created a MetaCookie+ system consisting of headset and aroma delivery system, which was able to track and alter the appearance and scent of a cookie in real time (Narumi et al., 2011). The researchers showed that, without changing the chemical composition of the food itself, 79% of participants experienced a change in the cookie taste using the pseudo-gustatory display. Going beyond taste perception, AR has also been shown to modify people’s level of perceived satiety by altering the apparent size of the food consumed using real time-shape deformation (Narumi et al., 2012).

However, such custom AR technology requires a high level of technical expertise in computer vision, making it relatively infeasible in most sensory testing situations. In the current study, we created a simple yet convincing mixed-reality VR setup which allows for the simultaneous presentation of food stimulus in both virtual and physical reality.

### Hypothesis Development and Contributions

To demonstrate how VR could be incorporated in flavor perception research, we decided to investigate a scenario which would be difficult to study in the real-world. Namely, we investigated the influence of visual appearance on the perceived flavor of black coffee. Since people have learned to associate a light brown color with milky coffee, we hypothesized that *a black coffee which appears to be light brown would be rated as tasting sweeter and creamier than one that appears dark brown*. If the hypothesis holds, then we could develop future applications where people can add “virtual creamer” to their coffee to cut down on calories while still maintaining the perception of creaminess. Moreover, this study is uniquely suited for VR because, in the real-world, it would be difficult^1^ to make black coffee appear light brown without altering its taste.

From a theoretical perspective, this study is a proof of concept for using VR as a way to study the merging of virtual and actual sensory cues in the formation of our eating experience. Notably, unlike previous VR studies which separated virtual and real sensory cues (e.g., Huang et al., 2019, where participants first saw a color cue and then tasted the samples in a black screen), the present study enhances the realism of the situation by enabling the participants to simultaneously interact with the same object in both the physical and virtual environment. From an industrial perspective, this study demonstrates the possibility of performing rapid product testing with a consumer panel, in situations when it may be time-intensive or costly to produce products with the same range of visual features in the real-world.

## Materials and Methods

### Participants

Thirty-two participants (5 women, 27 men) aged 18–38 years (*M* = 23.1, *SD* = 3.1) were recruited for the study from York St. John University. Participants were recruited at computer science labs on campus by word of mouth. The studies took place between 10 a.m and 6 p.m (13 participants took part before 1 p.m, 19 participants after 1 p.m). No further selection criteria were applied other than having normal senses of vision, smell, and taste. All participants gave their informed consent to take part in the study. The study was approved by the Research Ethics Committee of York St. John University.

### Coffee Samples

Black cold brew coffee (Califa Farms) were used in the study. To test sweetness level as a possible moderator in color–taste influences, two sweetness levels were created with the addition of either 4% or 8% of sucrose by volume. The coffee was served in 30 ml samples.

### Technical Setup

**Figure 1:**
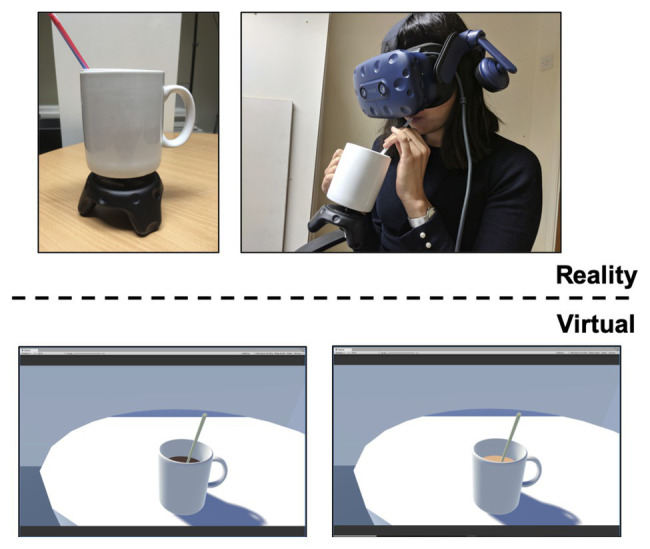
Experiment setup. A virtual reality (VR) tracker (HTC Vive) was attached to the physical mug so that the movement of the mug would be aligned in VR and in real life. The bottom images demonstrate the two color conditions (light brown, and dark brown) of coffee appearance in VR.

A room (4 m × 4 m × 2.3 m) with a table (0.9 m in diameter), mug, and straw was modeled in the Unity game engine (Unity Technologies) to mirror the physical setup in the experiment room (Figure 1). The color of the coffee was manipulated in VR such that it either appeared light brown or dark brown. A straw was placed in the mug so participants could consume the coffee while wearing the VR headset (HTC Vive Pro).

In order to create a mixed-reality environment where the mug of coffee would move synchronously in VR and the physical world, a VR tracker (HTC Vive) was placed under the physical mug. This way, the movement of the mug was aligned such that when participants lifted the mug in the physical world, the mug also appeared lifted in VR. such that the mug could be tracked in the VR environment as well.

### Procedure

A within-subject study with two virtually presented coffee colors (light brown, and dark brown) and two levels of sucrose in the physical coffee product (4, and 8%) was carried out. The study took place at a research laboratory at the Department of Computer Science at York St John University. Each participant was seated in front of a table. First, they read an information sheet and gave consent to partake in the study. Next, they rinsed their mouths with water and were instructed to wear the VR headset.

At the onset of each trial, the participant was instructed to pick up the mug from the table and take a sip from it. After tasting, participants were asked to orally evaluate their liking for the drink as well as its sweetness and creaminess on 1–9 scales (with 1 being not at all, and 9 being very much). These evaluations were reported verbally by the participants, and then recorded by the experimenter *via* a Qualtrics questionnaire. Participants then placed the mug back onto the table after giving their evaluation. In between trials, the experimenter switched out the coffee sample inside the mug. They also handed a cup of water to the participants, who rinsed their mouths with water between each trial. It should be stressed that participants wore the headset during the entire tasting session and never saw any of the actual coffee samples.

Each participant tasted all four color (light brown, and dark brown) and sweetness (4, and 8%) combinations. The order of conditions was counterbalanced across participants using a Latin Square Williams Design.

After tasting all four samples, participants removed their headsets and completed the rest of the Qualtrics questionnaire on a laptop. They answered questions regarding their VR experience (never used it before, < 1 h, 1–5 h, > 5 h), frequencies of drinking black coffee and of drinking milky coffee (daily, 4–6 times a week, 2–3 times a week, once a week, never), and preference for sweet and bitter foods (scale from 1 to 9).

Each experimental session lasted approximately 15 min and participants were debriefed afterwards.

### Data Analysis

Data from all 32 participants were included in the analysis. After checking for data normality and multicollinearity between the measured variables (i.e., liking, sweetness, and creaminess), a repeated-measures multivariate analysis of variance (RM-MANOVA) was conducted with coffee color (light brown, and dark brown) and sugar level (4, and 8%) as within participant factors (SPSS, version 25). The model included liking, sweetness, and creaminess as measures. While our sample size is too small for in-depth demographic analyses, we reported summary statistics of the participants’ VR familiarity, coffee drinking frequency, and preference for sweet and bitter tastes to communicate more information about the background of the participants.

## Results

To check for multicollinearity, Pearson’s correlation coefficients were calculated between measures of sweetness, creaminess, and coffee liking. It revealed, as expected, that coffee liking is positively correlated with perceived sweetness (*r*_128_ = 0.19, *p* = 0.034) and with creaminess (*r*_128_ = 0.26, *p* = 0.003), and that sweetness and creaminess are positively correlated (*r*_128_ = 0.35, *p* < 0.001). To better understand the drivers of coffee liking, we found that creaminess is positively correlated with liking even controlling for sweetness (*r*_125_ = 0.21, *p* = 0.019), whereas sweetness is not correlated with liking after controlling for creaminess (*r*_125_ = 0.11, *p* = 0.229). Sweetness and creaminess are positively correlated after controlling for liking (*r*_125_ = 0.32, *p* < 0.001).

**Figure 2:**
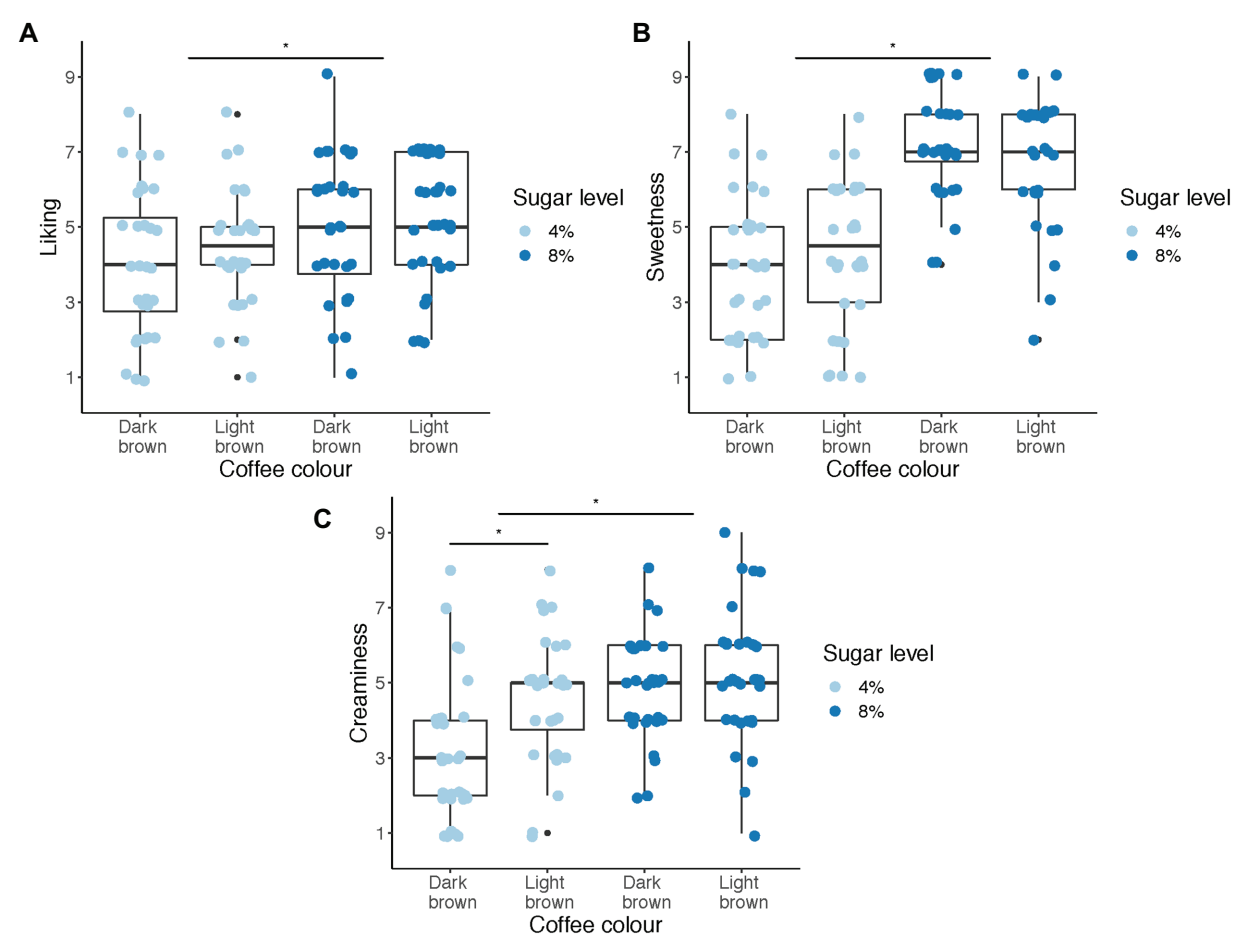
Scatter and boxplots showing participant ratings of coffee liking **(A)**, sweetness **(B)**, and creaminess **(C)** under both VR color conditions (dark vs. light brown) and for both sugar levels (4% vs. 8%).

Participants’ ratings for coffee samples in all conditions are shown in Figure 2. RM-MANOVA revealed a significant main effect of color [*F*(3,29) = 3.13, *p* = 0.04, *Wilks Lambda* = 0.76] and of sugar level [*F*(3,29) = 55.01, *p* < 0.001, *Wilks Lambda* = 0.15]. We did not observe a main interaction effect between color and sugar level [*F*(3,29) = 1.35, *p* = 0.28, *Wilks Lambda* = 0.88].

Univariate ANOVAs revealed a significant main effect of color on creaminess [*F*(1,31) = 9.48, *p* = 0.004, *η_p_*^2^ = 0.23], where the coffee was rated to taste 20% creamier when it appeared light brown compared to dark brown (*M_dark_* = 4.08, *SE* = 0.25, *M_light_* = 4.88, *SE* = 0.19, *p* = 0.004, *Hedges’ g_av_* = 0.62). Moreover, the color-induced change in creaminess was only observed for the 4% sugar-added coffee (*M_dark_* = 3.28, *SE* = 0.34, *M_light_* = 4.88, *SE* = 0.24, *p* = 0.010, *Hedges’ g_av_* = 0.87), and not for the 8% sugar-added coffee (*M_dark_* = 4.56, *SE* = 0.29, *M_light_* = 5.19, *SE* = 0.31, *p* = 0.348). In contrast, we did not observe an effect of color on liking [*F*(1,31) = 1.08, *p* = 0.306] or sweetness [*F*(1,31) = 0.003, *p* = 0.958].

In terms of sugar level, the 8% sugar-added coffee was liked more [*F*(1,31) = 9.92, *p* = 0.004, *η_p_*^2^ = 0.24] and rated as sweeter [*F*(1,31) = 134.93, *p* < 0.001, *η_p_*^2^ = 0.81] and creamier [*F*(1,31) = 20.22, *p* < 0.001, *η_p_*^2^ = 0.40] compared to the 4% sugar-added coffee.

An overview of the participants’ background revealed very limited VR familiarity, with 19 out of 32 participants reporting they have never used VR before, six having used VR for less than an hour, four having between 1 and 5 h of experience, and three having over 5 h of experience. Black coffee drinking frequency was also very limited, with 27 out of 32 participants reporting never drinking coffee black, two reporting drinking black coffee once a week, one reporting 2–3 times a week, and two reporting daily consumption. Milky coffee drinking frequency was higher, with 14 out of 32 participants reporting never drinking milky coffee, five reporting drinking once a week, four reporting 2–3 times a week, three reporting 4–6 times a week, and six reporting daily consumption. In terms of food preference, preference for sweet foods (*M* = 6.66, *SD* = 1.70) was significantly higher than preference for bitter foods [*M* = 4.44, *SD* = 1.70, *t*(31) = 4.48, *p* < 0.001].

## Discussion

The results of the present study demonstrate that color cues from VR and gustatory cues from the real-world may be integrated to influence creaminess evaluation of black coffee, with coffee that appeared to be light brown rated as significantly creamier compared to coffee that appeared dark brown. This result is in line with previous evidence of color-gustatory bias (see Spence et al., 2010, for a review), but with the novelty of using VR to assess food stimuli that are otherwise difficult to set up physically.

Given the changes observed in creaminess when coffee color was changed in VR, the question naturally remains whether we might expect a similar degree of change if the color of the coffee were physically altered in real life. As mentioned in the introduction, there has been a body of studies demonstrating consistency between consumer ratings and behavioral tasks performed in real life vs. in VR (Persky et al., 2018; Ung et al., 2018; Siegrist et al., 2019; Sinesio et al., 2019; Ammann et al., 2020). Therefore, we have some confidence that our findings in VR would translate to a real-world experience. Moreover, it might be possible to validate our findings in real life in the future, by either making light brown coffee appear darker with food coloring, or, using high-concentration white food coloring (unbeknownst to the authors at the time of conducting the research originally) to lighten black coffee. Granted, both methods would require extensive pre-testing to ensure that no additional flavors or textures are introduced by the food coloring. That said, we can compare our current findings with previous studies showing the effect of beverage color on rated taste intensities in sucrose solutions or in fruit beverages (e.g., Johnson and Clydesdale, 1982; Clydesdale et al., 1992; Lavin and Lawless, 1998; NDom et al., 2011). Moreover, in terms of coffee-specific results, while we are not aware of any studies manipulating coffee color (without changing its components), there is evidence demonstrating that changing the color of the coffee cup can influence the taste of the coffee (Van Doorn et al., 2014; Carvalho and Spence, 2019). As seen in Table 1, our effect size (*Hedges’ g_av_* = 0.62) is roughly in line with the range of effect sizes observed in previous studies involving the manipulation of color, although we should qualify that this is only a very approximate comparison, since none of the color-bias studies analyzed have measured mouthfeel.

**Table 1:** Effect size analyses of previous studies demonstrating the effect of color (either food-intrinsic or extrinsic) on taste perception.

Study	Design	N	IV	Food-intrinsic or extrinsic color	DV	Effect size
Present study	Within-subjects	32	Coffee color (light or dark brown)	Intrinsic	Creaminess	Hedges’ g_av_ = 0.62
Carvalho and Spence (2019), study 1	Between-subjects	82	Cup color (white or pink)	Extrinsic	Sweetness	Hedges’ g_s_ = 1.17
Carvalho and Spence (2019), study 1	Between-subjects	82	Cup color (white or pink)	Extrinsic	Acidity	Hedges’ g_s_ = 1.27
Lavin and Lawless (1998) study 1	Within-subjects	74	Fruit beverage color (light or dark red)	Intrinsic	Sweetness	Hedges’ g_av_ = 0.30
Lavin and Lawless (1998) study 1	Within-subjects	74	Fruit beverage color (light or dark green)	Intrinsic	Sweetness	Hedges’ g_av_ = 0.22
NDom et al. (2011)	Between-subjects	24	Fruit beverage color	Intrinsic	Taste	Hedges’ g_s_ = 0.83

In order to compare between effect sizes of between and within subjects studies, we calculated Hedges’ g_s_ (between-subjects design) and Hedges’ g_av_ (within-subjects design), as recommended by Lakens (2013). We could only calculate effect size for those studies where either t-values or the combination of means and standard deviation/error were reported.

Furthermore, it is important to note that the color-induced change in creaminess evaluation was only observed for 4% sugar coffee and not for the 8% sugar coffee. There are several plausible reasons for why we did not observe any color-induced changes in creaminess in the 8% sugar coffee. Since sweetness and creaminess were highly correlated, it is possible that participants could have experienced a ceiling effect where the 8% coffee was rated as creamy even in the dark-brown VR condition. This is especially plausible since higher sugar level could increase perceived creaminess by increasing the viscosity of the coffee (Frøst and Janhøj, 2007; Wagoner et al., 2019). Alternatively, there might have been a contrast effect, whereby the 8% sugar coffee did not match consumer expectations in the dark brown VR condition, and disconfirmation of expectations resulted in a higher creaminess rating in the dark brown condition than participants would have otherwise given (Piqueras-Fiszman and Spence, 2015). Regardless, the fact that color-creaminess effects are dependent on the product itself demonstrates that participants’ creaminess ratings were driven by more than just color cues.

Moreover, while we observed a color-induced change in creaminess, altering coffee color in VR did not significantly alter perceived sweetness or liking. This is possibly because, in everyday life, coffee that appears light brown, i.e., milky coffee, is not necessarily also sweetened. Therefore, participants had no reason to associate a lighter brown color with sweetness whereas milky coffee is more clearly associated with creaminess. Furthermore, as we did not apply strict selection criteria commonly used in sensory studies (e.g., asking participants to fast or avoid eating strongly flavored foods 2 h before the study), it is possible that we could have obtained more precise results had these stricter guidelines been followed.

From a methodological view, the experimental procedure outlined here can be easily applied in psychological research as well as market research and new food research and development. Through the use of a commercially available HTC Vive tracker, we were able to create a heightened sense of reality in VR by enabling participants to simultaneously see the food in VR while touching and tasting it physically. This goes one step beyond previous research in VR eating experiences, which either do not include a model of food in VR (e.g., Sinesio et al., 2019), or if they do, only provide a static model of the food that does not track the motion of the physical food in the real-world (e.g., Huang et al., 2019). Our research also extends previous AR experiences, because our relatively simple technological setup is easily accessible [compared to the deep learning visual learning algorithms used in Ueda and Okajima (2019)] and offers a range of motion [compared to limited to the space of the projective systems as in Nishizawa et al. (2016)]. Furthermore, the strength of our system lies in using real foods in a mixed reality setup, since the digitalization of chemical input, such as shown by The Virtual Lemonade or Vocktail (Ranasinghe et al., 2017a,b) is still far from convincing (see Spence et al., 2017, for a review). To summarize, in terms of psychological research, we can use this system to study multisensory integration by presenting tailored combinations of digitally introduced audiovisual information and physically introduced chemosensory information. From an industry perspective, this VR system would enable the rapid testing of product and packaging visual appearances, without having to produce the same range of visual features in the real-world. Furthermore, we can use the VR system to evaluate the relative influences of product, packaging, and environmental features on consumer food perception, preference, and eating behavior.

Nevertheless, we should point out that the study has several limitations. One obvious limitation is the relatively small sample size tested (*N* = 32), although this is in line with previous VR and color studies [*N* = 50 per cell (study 1) and *N* = 25 per cell (study 2) in Ammann et al. (2020); *N* = 41 in Chen et al. (2020)]. Another demographic issue is the admittedly uneven gender distribution of mostly men, due to the fact that we collected a convenience sample from computer science courses. Therefore, it is unclear to what extent our findings on coffee color and creaminess can be generalizable to the wider population.

Furthermore, a limitation of the current study is that we only recorded the participants’ subjective coffee evaluations, which could have been influenced by response bias and demand effects. That said, the fact that we observed different color-creaminess effects with different sugar levels suggests an expectations effect (Piqueras-Fiszman and Spence, 2015) where color cues only played a role when the coffee was not already perceived as creamy due to its high sugar content. In the future, VR-food studies can be improved by collecting behavioral data, such as discrimination testing or drinking speed/quantity, to get a better understanding of color-induced perceptual effects. Another idea is to simultaneously collect biometric data such as electrodermal conductance, heart rate, or electroencephalography (EEG) to better understand the participants’ emotional experience while simultaneously interacting with food in the virtual and physical world.

Thinking more broadly, a variety of research projects already use VR to create facsimiles of infeasible or impossible situations, often for therapeutic purposes, in order to better understand how people react to these situations. For instance, VR in widely used in the treatment of eating disorders in the form of exposure therapy (see Clus et al., 2018, for a review), by presenting virtual food stimuli or by altering the patients’ own body image. Our novel methodology of simultaneously presenting both digital and physical food cues therefore introduces a way for researchers to combine audiovisual information coming from digital sources with chemical information coming from the food itself in the physical world. In other words, this combination makes it possible for participants to see one thing while eating another, all the while believing they are eating what they are seeing. Some future applications of this technology could be to enable multisensory eating scenarios whereby participants could reduce their sugar/salt/fat intake with “virtual seasoning” (see Wang et al., 2019, for some ways to enhance sweetness without adding sugar), or learn to familiarize themselves with new foods. For example, VR may be a way to introduce more vegetables for children by changing the visual appearance to be more similar to those of more acceptable foods, such as suggested by Petit et al. (2019) for baby carrots to appear like French fries. Of course, any such research is predicated on first acquiring an understanding of whether longer term use of this VR method would lead to sustained changes in food-based perceptions, and how best to transition from experiencing foods with VR into lasting “real-world” food preferences and behaviors. Nevertheless, in the context of the increasing popularization and accessibility of VR technology, this research could therefore be a first step towards using VR in combination with eating scenarios to encourage healthier eating behavior and more sustainable food choices in the general population.

## Data Availability Statement

The datasets presented in this study can be found in online repositories. The names of the repository/repositories and accession number(s) can be found at: https://osf.io/etu59/.

## Ethics Statement

The studies involving human participants were reviewed and approved by Research Ethics Committee of York St John University. The patients/participants provided their written informed consent to participate in this study.

## Author Contributions

QW: Conceptualization, methodology, formal analysis, visualization, and writing – original draft preparation. RM and SW: Investigation, writing – review, and editing. DZ: Conceptualization, methodology, software, writing – review, and editing. All authors contributed to the article and approved the submitted version.

### Conflict of Interest

The authors declare that the research was conducted in the absence of any commercial or financial relationships that could be construed as a potential conflict of interest.

## Footnotes

^1^Although high-concentration white food coloring apparently exists, which might make this scenario easier to test in the real world.
